# Periscope

**Published:** 1881-10

**Authors:** 


### PERISCOPE.

### Medical Advance, May, June and July.

Prof. Owens publishes an interesting paper, which is, to all intents and purposes, a plea for a pathological view of materia medica. To some of his sentences we must take exception. For instance, he says, "Hahnemann speculated, and then demonstrated in therapeutics." This is a mistake. Hahnemann *experimented* and then demonstrated in therapeutics. In other words, he followed the inductive method. By this method the scientific inquirer does not *speculate.* On the contrary, he banishes all theory from his mind, and, by the process of experiment, "addresses a question to nature," pure and simple. Free from all theoretical bias, the mind of the investigator is prepared to accept the true answer, no matter how unexpected the character of it. This was the method of Hahnemann, and it is to be particularly borne in mind that Hahnemann availed himself of the inductive method at the very time it became established by the eminent workers in physical science. At the very time that Hahnemann was making his drug experiments Sir Humphrey Davy was applying electrolysis to determine the nature of the alkalies and alkaline earths. No speculation was used by Davy in his experiments. Neither was any used by Hahnemann in his investigations. "No man ever took a step forward in anything," says Prof.

Owens, "unless he speculated, conjectured and formed hypotheses." The objection to this seems to us to be that it is in accordance with the processes of the old alchemists, who first formed a hypothesis, and then undertook to prove it by experiments (the *deductive* method). Hence they were always blundering. Moreover, it is the method in the old school with regard to drug therapeutics to-day. Hence they are so empirical. Hence it is that no idea in drug administration, however crude, is too absurd for the average doctor to reject. We think that it is in Boswell's Johnson where it is stated that a physician told an ænemic young girl that in order to be *strong* she must take *iron;* not that of the drug stores, but the *steel filings* of the machine-shop, a teaspoonful three times a day! Thus, because steel is *strong*, it must *strengthen* the human system. Does not this idea exist in the mind of the old school doctor to-day? We think it does. Tanner's "Index of Diseases and their Treatment" (Henry Renshaw, London, 1866), constantly recommends preparations of STEEL. Now such follies as this come from the habit of speculating. Homœopathic principles appear doubly absurd to an old school doctor because he falsely assumes them to be the result of speculation, and his inferences in that regard are strengthened by the thoughtless remarks of its followers. Hence Prof. Owens will see why we take him to task.

Dr. McNeil reports two cases of eczema vesiculosa. In the first case, the patient had had *rheumatic pains with stiffness, worse by rest; better from motion.* Cured with *Rhus tox.* The second case was attended with *itching from getting warm; burning of feet, so that they must be put from under the bed-cover. Sulphur* 500, then 6 M, then 55 M, then 81 M cured.

A case of persistent vomiting of pregnancy was relieved by *Sulphur.* The specific indications were: *red lips; everything eaten turns sour; hot feet, must put them out of bed.*

A case of pernicious intermittent fever exhibited the following symptoms: *Feeling as if contents of abdomen were pressed upward; pains all over, worse from motion; faintness on rising. Bryonia* cured.

Dr. McNeil's comments: "It is a prevailing delusion in our school that in a case of pernicious intermittent, nothing but massive doses of quinine will prevent a return of the paroxysms—the third return of which, they say, is certain death. That is all nonsense."

"I believe that quinine frequently, in the climate in which I live, changes benignant intermittents to malignant ones." "Cholera, yellow fever, typhus and diphtheria have given homœopathy a fame which she can only lose by the recreancy of her professed followers." Respectfully referred to the philosophers of the pestilential Wabash.

Dr. Nash reports a case of ulceration of uterus, with constriction of os, and suppressed menses. Symptoms: *pain and weakness in back; flushes of heat; weak and faint after they pass off; great heat of top of head; feet cold all day, but burn at night; must put them out of bed*; *restless and sleepless at night.* *Sulphur* CM (Fincke) cured.

Dr. Nash then indulges in some caustic remarks at the expense of an eclectic who "feared some homœopath would 'cudgel' him" for his treatment of a similar case, reported in January number of *Advance.* He used topical applications "just as an allopath would use them." "What is most to be regretted," says Dr. Nash, "is that professed homœopaths are so often, of late, resorting to old school, antipathic measures, and then trying to defend themselves on the ground that it is rational and scientific."

Dr. Gilbert reports a case of threatened abortion prevented by *Gelsemium* CC. Indications were: *fright caused a pain in uterus; worse from retching, followed by vomiting. The pain extended up to back of head.*

Two cases of enuresis nocturna were benefited by *Lachesis.* The indication was derived from the symptom: *this remedy seems to profoundly affect the voluntary system, and the will-power must supply the deficiency.*

### Medical Advance, June.

Dr. Gillard reports a case of ulceration of descending colon and rectum cured, after a three years' struggle, with *Lyc.*, after many other remedies had failed. The indications are a little obscure, but the general statement of the case is very interesting.

S. L. reports a case of tertian fever, attended with dry, teasing cough, for which *Rhus tox.* 30, was prescribed on Dunham's indication, "*a dry, teasing cough before the chill, continuing after the chill comes on.*" Cured.

A case of pneumonia *caused by getting wet*, and having the symptom, *every bone in the body aches; bruised feeling all over;* received *Rhus tox.* 30 M. Cured.

A case is reported of nasal catarrh, having thick, greenish-yellow discharge. Cured by *Syphilinum* after other remedies had failed.

A case of gonorrhœal rheumatism was cured with *Medorrhinum*, CM. Unfortunately, the indications for these remedies are not very clear, so that the giving of them is almost empirical. Neither are yet sufficiently well proved for clear use.

Dr. Owens was called to a case of suspended digestion, with symptoms so violent that the patient was in imminent danger of death. He had eaten heartily of buckwheat cakes for supper. The indications were: *must have the doors and windows open to get breath; profuse cold sweat all over, and standing in large drops on head and face; engorged stomach, sickness and nausea; white and glassy eyes; labored respiration. Puls.*, 6 M, cured; relief commencing in four minutes. The doctor says he prefers the *simillimum* to a stomach pump. This will horrify the eclectics.

An old man, who had been "raising thick, light-colored, tenacious, nauseating mucus," to the amount of two quarts in twenty-four hours, presented the following indications: *discharge of yellowish pus from the left eye; eyelids sticking together in morning after sleeping; discharge of hot water from the eyes; burning in the hands and feet. Sulph.*, 10 M, relieved. Then dryness of mouth and mucous discharge from rectum followed. *Carbo v.* was given, and later *Sulphur* again. Symptoms all cured.

A young lady had *sharp, cutting, grinding pains in bowels before menses, causing sickness and nausea. Gelsemium* cured.

A minister had neuralgia of the stomach, for which he always took large doses of *Morphine. Nux*, 50 M, cured.

### The Homœopathic Journal of Obstetrics, May.

Dr. Guernsey reports a very interesting case of retained placenta, after an abortion, at the fourth month. The symptoms were: *abortion from contusion, sensation of soreness as from a bruise; hot head and cool body; great restlessness, wishing to change her position constantly.* These being indications for *Arnica*, that remedy was given, and in forty-eight hours the placenta came away spontaneously.

The above is in striking contrast to some other cases reported in this same journal, where the operator, though professing to be enlightened by the principles of the new treatment, nevertheless continues the bad practice of tearing away piece-meal the retained placenta (even though it requires three days to do it), just like an old-school doctor. Yet, according to these scientific doctors, it is blundering practice to wait so long upon the action of the indicated remedy.

### U. S. Medical Investigator, May and June.

A "beginner in homœopathy" asks if it be true that medicines will change the mal-position of a fœtus in utero. The editor answers by reprinting a paper of the late Mercy B. Jackson, M. D. relating fourteen cases in her practice so changed by the administration of *Pulsatilla.*

Dr. Johnson reports a cure of constipation with *Opium*, 200; the characteristic being a *scanty stool of small black balls.*

Dr. Oertal mentions a case of Hæmaturia, where the *urine was passed in drops, with great pain. Large coagula of blood passed out with much pain. Cantharis*, 30th, relieved very much. It was succeeded by several other remedies according to indications, and at the end of four months, the patient was cured.

Dr. Tuckey reports a case of angina pectoris, with sensation of a ball rising from the heart to the throat, threatening suffocation. *Lachesis*, 6th, cured.

Another case was much relieved by *Ignatia*, and ultimately cured with *Amyl-Nitrite*, 1 x.

A case of headache, with *throbbing pain, languor and heaviness, pain in eyes, with blurred vision*, was cured with *Bell.*

A case of painful menstruation, preceded by diarrhœa, first *watery and burning*, followed by blood and mucus, and *tenderness of abdomen*, was greatly relieved by *Arsenicum.* Later, *Colocynth.* produced a cure.

A case of gastritis, of three years' duration, showed the following symptoms: after taking food, there was great sickness and violent retching, lasting several hours, until vomiting occurred, which relieved; tenderness over the stomach; distention after eating; yellow fur on tongue; mouth dry and parched; coppery taste. *Cuprum*, 6th, cured.

A man, tainted with syphilis, had deep suppurating "serpiginous" ulcerations upon the back, cured with *Kali-iodium* in two weeks.

Dr. Tilden writes upon the cause of pigeon-breast in children, and shows that it is largely due to the habit of lifting children, by placing the hands upon the chest beneath the infant's arms. This causes compression of the ribs, and permanent deformity when persisted in. The proper way to lift a child is to place one hand under the buttocks, using the other to steady the body.

### Medical Call, July.

Dr. Whipple gives the "best treatment" for "rheumatism or any other disease, not of a surgical or chemical nature." It "is to select your remedies in each case in strict accordance with *the* law of therapeutics, as expressed in the formula, '*Similia Similibus Curantur.*'" He then proceeds to illustrate this position. A laborer, with inflammatory rheumatism, exhibited the following symptoms: *profuse perspiration, but giving no relief, but rather aggravating the weakness; dirty yellow coating of the tongue; thirst, restlessness, anorexia, nightly aggravation, especially in the warm bed. Merc. Sol.*, 30, cured in six days.

A woman, being taken down with same disease, and showing the same symptons, received the same medicine, and was fully cured in six days.

In another case of rheumatism, *the pains were worse on first beginning to move, better from continuing to move. Rhus tox.*, 30, cured in a few days—even curing a "double lateral curvature of the spine," which had been caused by the pains.

The doctor will not say that he *never* allows topical applications in such cases, as "it may be necessary in order *to satisfy the patient and friends.*" This prescriber is evidently a genuine homœopathist. It is pleasant to meet with such, and to report their cases.

Dr. Parkhurst cured a case of rheumatism with *Apocynum;* the indication being, *the patient screams if any one approaches her, or points the hand at her.*

Dr. Hallett cured a case of epileptiform spasms, of five years' duration, with *Puls.*, the indication being, *profuse secretion of tears.*

Dr. Wakefield reports two cures of diarrhœa and vomiting in children with *Calc-c.*, the indication in each case being *vomiting of milk in curds; thin whitish diarrhœa* (green in one case); *does not sleep after* 3 A. M.; *profuse sweat of the head, wetting the pillow.* One of these cases was followed by a painful tumor in the hip, which was prevented from suppurating by *Hepar*, 30.

Dr. Campbell cured two cases of tobacco amaurosis with *Nux-v.*, 3.

Dr. Burnett cured a stubborn cough with *Aralia;* the indication was: *fit of asthma on lying down.* In three other cases the indications were: *cough coming on after first sleep*, waking the patient. *Aralia* cured.

### Medical Counselor, May.

Dr. Leonard reports a case of articular rheumatism, in which the indication first appearing was: *pain greatly aggravated by washing the hands in cold water;* also, *at night. Calc-carb.* partially relieved. The pain in shoulders still continuing, *Puls.* was given for the following symptoms: *numbness in left arm, from elbow down; mild, tearful disposition; sanguine temperament.* This was followed by a cure, and now the doctor contends that *Puls.* should have been prescribed at first.

Dr. Heath reports delivery of a child, where the breech presented, with a pair of Comstock's Cephalic Forceps, applied over transverse diameter of the pelvis.

